# ‘A lightbulb moment’: carers’ experiences of behavioural symptoms in motor neurone disease before and after MiNDToolkit

**DOI:** 10.1186/s12883-024-03746-5

**Published:** 2024-07-09

**Authors:** Eneida Mioshi, Sue Heal, Thando Katangwe-Chigamba

**Affiliations:** 1 School of Health Sciences, University of East Anglia, Queen’s Building Norwich Research Park, Norwich, NR4 7TJ UK; 2 Norwich & Waveney Branch, MND Association Norfolk, Norwich, UK; 3 Norwich Clinical Trials Unit, Norwich Medical School, University of East Anglia, Norwich, UK

**Keywords:** Amyotrophic Lateral Sclerosis, Frontotemporal Dementia, Carers, Caregivers, Behavioural symptoms, Internet-based intervention

## Abstract

**Background:**

To explore carers’ experiences of behavioural symptoms in Motor Neurone Disease (MND), before and after using the MiNDToolkit, a novel internet-based psychoeducational intervention to support management of behavioural symptoms (BehSymp) in MND. The study also investigated carers’ views and acceptability of MiNDToolkit.

**Methods:**

A qualitative process evaluation of carers engagement with, and acceptability of, the MiNDToolkit conducted using semi-structured interviews with carers (*n* = 11). All interviews were audio-recorded, professionally transcribed verbatim and analysed thematically.

**Results:**

Five themes were identified: (1) In the dark: carers’ experiences and reactions to BehSymp; (2) Others can see: the role of HCPs in identifying symptoms – and perceived opportunities for carers to receive support; (3) Shedding light: carers implementation and perceived impact of the MiNDToolkit content; (4) Acceptability and carers’ engagement with MiNDToolkit; (5) Future implementation. Carers’ experience of BehSymp was particularly distressing when symptoms were apparently out of context. MiNDToolkit appeared to support learning that BehSymp were part of MND. Content resonated with carers, who reported learning about the full picture of MND, which led to acceptance and use of newly learned strategies. Engagement with the platform was good, with varied input from HCPs. Greater and nuanced involvement from HCPs seem important to support management of BehSymp. Recommendations for a full-scale trial emerged, including adding a paper booklet to accompany the intervention and creation of new modules on emotional lability, changes in relationships, and transitioning to a care home.

**Conclusions:**

MiNDToolkit was acceptable to carers overall. Recommended improvements should be actioned in a full-scale trial.

## Introduction

Caring for someone with Motor Neuron Disease (MND) is multifaceted and emotionally demanding; care provided is varied and progressively more intense, including need to understand the progression of the disease, managing competing tasks and external resources and trying to maintain normality [1]. This is because MND is a progressive neurodegenerative disease characterised by degeneration of upper and lower motor neurons, leading to muscle atrophy, loss of function [2] and death. As such, people with MND (pwMND) become progressively dependent on others due to the deficits associated with the changes in various body systems [3]. Most of the care is provided by family members, usually a spouse or child [4], with or without the help of paid carers or others. Despite the major role that family members have in providing care in MND, there is very limited evidence on how to best support carers of PwMND [5], in particular when complex symptoms associated with frontotemporal dementia (FTD) are present.

The existence of behavioural symptoms (BehSymp) in MND, such as disinhibition, apathy, obsessive behaviours, and hallucinations, have been described for many decades [6]. However, MND’s link with FTD has been reignited more recently with the identification of the role of the C9orf72 gene [7, 8], which brought MND and FTD alongside a clinical, pathological, and genetic continuum. Since then, the development of assessments to support identification of cognitive and BehSymp has strengthened, and the international ALSFTD diagnosis criteria has been revised [9]. Efforts have been made to ensure regular assessments are included in MND clinical care, but these are still not consistently conducted in many specialist MND services, though healthcare professionals (HCP) show increased awareness [10]. Assessments of non-motor symptoms are of great relevance for not only for disease management, but especially for carers. BehSymp have been shown to be associated with carers’ perceptions of loss [11], and BehSymp are recognised to underpin higher rates of carer burden in many countries [12–15].

Evidence on clinical management of BehSymp is nascent. A single-centre Danish study investigated an online palliative rehabilitation blended learning program, with group peer-support and videos for carers of pwMND with BehSymp and cognitive deficits. Carers had good engagement with the intervention but low completion rates [16]. Of note, the intervention did not seem to offer specific support for behavioural symptoms. A recent randomised feasibility trial of the MiNDToolkit showed promising results [17] in England and Wales, and strong relevance for HCPs [18]. As such, the present study aimed to investigate MiNDToolkit’s acceptability by carers. This study was conducted alongside the MiNDToolkit feasibility trial. Here, we undertook a qualitative process evaluation to explore and understand carers’ acceptability and application of the MiNDToolkit intervention, and their experiences of behavioural symptoms prior to using MiNDToolkit, and after.

## Materials and methods

### Design

A qualitative process evaluation of carers’ engagement with and acceptability of the MiNDToolkit conducted using semi-structured interviews [19]. Ethical approval: London Queen Square Research Ethics Committee (19/LO/0692, IRAS260290).

### Study setting

The MiNDToolkit study (ISRCTN 15,746,123), a randomised controlled feasibility trial, was conducted across 11 sites in England and Wales between July/2021-March/2023. All participating sites including Hospitals, MND Care and Research Centres, Community teams and Hospices, had advanced MND care set up.

### The MiNDToolkit

MiNDToolkit is a complex intervention for the management of behavioural symptoms by carers [17]. The intervention consists of tailored online psychoeducational modules and strategies to manage reported symptoms. Learning and strategies are reinforced by trained HCPs during appointments.

### Participants and recruitment

Eligible carers (family carers, relatives or live-in professional carers) had regular contact with a person diagnosed with MND-FTD or MND with cognitive or behaviour impairment. 29 carers from England and Wales took part in the feasibility study [20]. Carers allocated to the intervention arm who completed the study (11/14) or allocated to intervention after the control period (former controls, 7/15), were invited to take part in the mixed-methods evaluation. The process evaluation results for HCPs have been reported elsewhere [18].

### Data collection

Eleven individual semi-structured interviews were conducted. Carers were asked about their understanding and experiences with symptoms of MND, and how they managed them before MiNDToolkit. To further explore engagement and acceptability, carers were then asked about their experience of the MiNDToolkit Online Platform and HCP support. Carers were also asked about their perceived impact of the intervention on the management of symptoms. Interviews were guided by a topic guide and conducted virtually (TKC) on MS Teams, audio recorded, and professionally transcribed verbatim (Topic guide, Appendix-1).

### Analysis

Interview data were inductively analysed using Reflective Thematic Analysis [21] to understand carers experiences. The analytical process involved an initial familiarisation with the data and making analytical notes followed by inductive coding by two researchers (EM, TKC) using NVivo. This was followed by discussion and analysis meetings where a former carer of a spouse with MNDFTD (SH) joined the two researchers (EM, TKC) to develop an understanding of primary concepts from the data and explore interpretations to identify key subthemes and themes, and validation of the findings. To enhance trustworthiness and interpretive validity of our findings, (1) we presented initial themes in an accessible format to some of the participants (as part of our study symposium), where participants were able to identify their experiences in the synthesized themes; (2) in addition, data analysis and synthesis was by a team which included a carer (SH) with lived experience, who was also able to identify their experiences within the data and was vital in interpreting the data from a carer’s point of view [22].

## Results

Eleven carers were interviewed between May 2022 and June 2023. Demographic and engagement in the intervention are described in Table 1.

**Table 1:** Carers’ characteristics and details of their engagement with MiNDToolkit (*n* = 11). *Please note the full sample of the MiNDToolkit study is reported elsewhere* [20]

Characteristics	
**Carer age (Mean and SD)**	63.18 (6.42)
**Carer gender**	9/11 female 2/11 male
**Relationship with the PwMND**	10/11 spouse or partner 1/11 parent
**Hours of care provided per week**	
1–4 h/week	3/11
15–22 h/week	1/11
31–49 h/week	4/11
50–99 h/week	2/11
100 h or more/week	1/11
**Diagnosis given to the person with MND, as reported by the carer**	
ALS	4/11
Bulbar onset or Progressive Bulbar Palsy	2/11
Progressive Muscular Atrophy Primary Lateral Sclerosis	1/11 0/11
MNDFTD	3/11
Not sure	1/11
Behavioural symptoms (MiND-B, total max 36)*	Median 24 (IQR 17, 28)
Apathy (% score)**	58.33
Disinhibition (% score)**	56.25
Rigidity and perseverative behaviour (% score)**	62.50
Engagement with MiNDToolkit	
Engagement with the MiNDToolkit platform (3 month-period) *Number of times logged in during intervention phase (Mean and SD)*	20.18 (9.65)
Initial allocation of study arm	10/11 Intervention 1/11 Control
Carers asking to use the MiNDToolkit beyond intervention or Control period	8/11
HCP reinforcement	Yes, 7/11 Online only, 4/11

*MiND-B total raw higher scores denote fewer behavioural changes. Cut-off is 32

**Subscores were corrected to a percentage to allow for clinical comparison between behavioural domains. After conversion, lower percentage reflects more pronounced behavioural symptoms

**Fig. 1:**
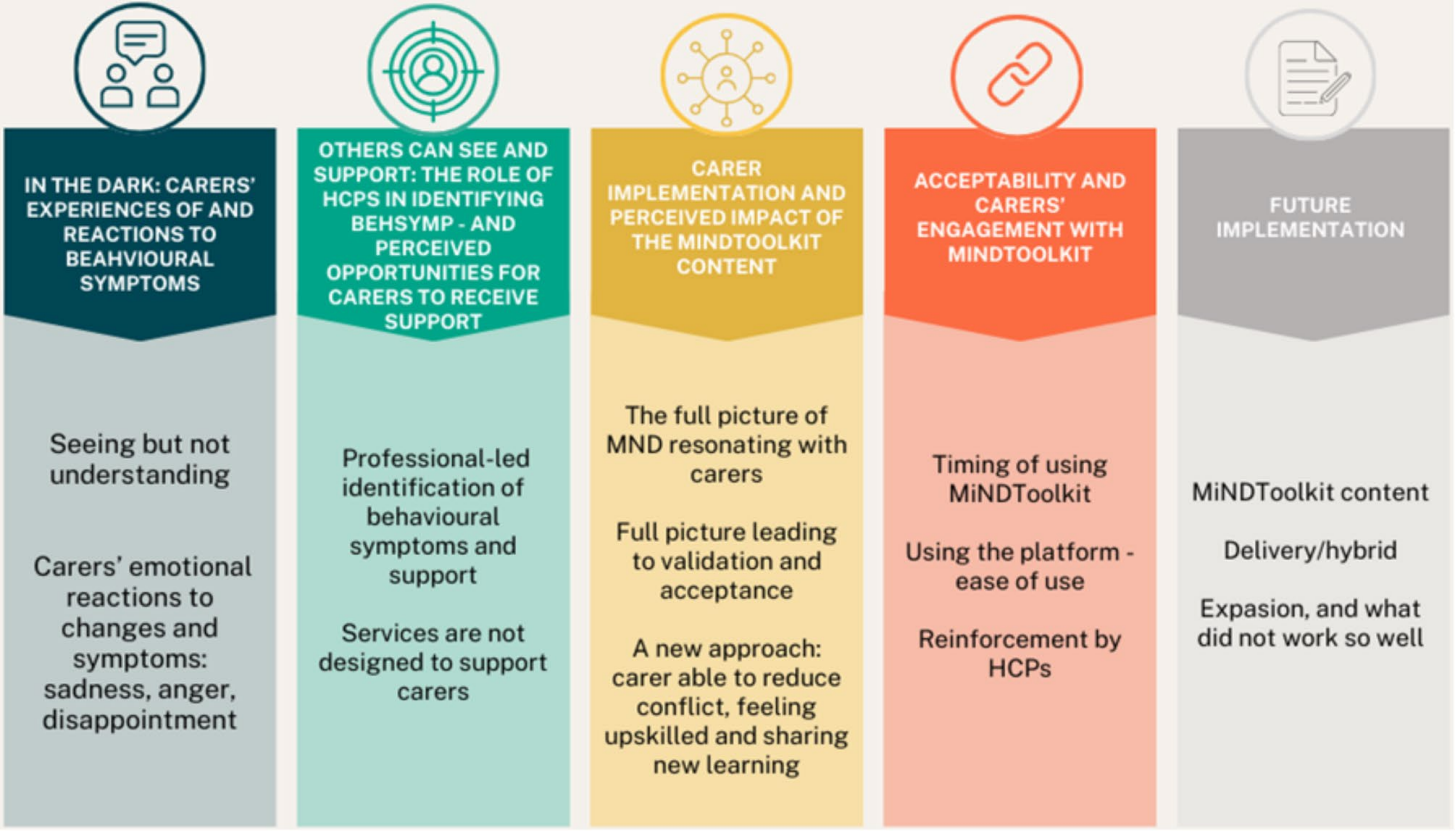
Themes and sub-themes identified in carers’ interviews exploring experiences of behavioural symptoms before and after MiNDToolkit

Five themes were identified (Fig. 1).

### Theme 1. In the dark: carers’ experiences of and reactions to behavioural symptoms (Fig. 1)

#### Seeing but not understanding

Carers’ recollections and experiences of when BehSymp were first noticed in relation to the time of diagnosis varied, with some first noticing the symptoms prior to diagnosis, while others after diagnosis was made. Carers gave examples of a range of behavioural symptoms: inability to understand another person’s perspective, lack of insight, rigidity, eating changes, and hallucinations.

*“Well I didn’t actually associate it with MND. This was quite a while before he was diagnosed. He started having an obsession that we had bedbugs in the house and that he had been bitten. Anyway, I spoke to his GP about it […] and the GP arranged to come up and see him. They had given him some [inaudible] cream which takes away the itching and the sensation that he’s got, bugs crawling on him, and that seems to have done the trick. But every now and again he will still bring it up. He might have a nail scratch that, you know, he’s scratched himself during the night and then he’ll say, ‘Look, I’ve been bitten.’ And he’s actually produced things in his hand and said, ‘Look, I’ve caught one,’ and it’s been a piece of fluff*.” ***Carer 02 describing an example of hallucination***.

***“****An example is like if we’re both getting dressed and he’s dressed, he’ll want to put his coat on long before we’re ready to go out and he expects me to stop what I’m doing to come and put his coat on, even though he’s not ready. Or if he’s looking for something, or if he wants his nails done and I’m doing something and I stop whatever I’m doing to attend to it, that’s what I mean by that selfishness. Well, he would have never done that before, he has no consideration for whatever I could be doing”.****Carer 01 describing an example of rigidity***.

*“I mean I would make, I make home made soup but when he was having soup every day, he wouldn’t eat it, he would insist on having a shop bought soup, not least of all because he wanted to know how many calories were in everything he ate.”****Carer 09 describing an example of eating changes***.

*“He gets obsessed about silly things like he thinks the next door neighbour put a new fence up and he’s convinced, even though he’s housebound, he’s convinced that that neighbour has stolen land and moved the fence closer onto our property.”****Carer 02 describing an example of delusion***.

A shared experience reported by most carers was the inability to link the BehSymp to MND. Carers reported observing behaviours that did not make sense or were out of character. Although the changes were noticeable, carers could not understand the reasons behind unusual reactions and behaviours from the PwMND. This lack of understanding led to carers’ confusion, and constant attempts to try to make sense of what was happening.

*“The changes in the personality was there, in behaviour, but then I thought it was, I put it down to other things, i.e., maybe depressed or there were times when we were socialising with friends and he would drink. And he normally, he would drink and it’s just the way it would affect him, and so I thought, “Oh my God, there is a problem with the drinking.” But there wasn’t because I think it’s just whatever was going on.” So before the diagnosis, I knew that something was wrong but I couldn’t put my finger on it.”****Carer 01***.

Reflecting back, some carers noticed that physical deficits associated with MND affected the expression of behavioural symptoms, e.g., when bulbar symptoms were present and inability to verbally communicate disguised symptoms.

*“He’s always been very philosophical. I think at the moment communication is very difficult because there’s no speech but he uses a phone with an app on it, predictable app, and various other things to help him communicate. But at the moment because the cognition isn’t so good and because the fine motor skills are not so good, typing and reading have become very difficult. In fact he can’t read at all and he can’t write, he’s lost his fine motor skills, so in his hands.”****Carer 03***.

#### Carers’ emotional reactions to the changes and symptoms: sadness, anger, disappointment

The lack of recognition of behavioural symptoms made carers feel alone, as they did not want to share their fears and worries with the PwMND or felt they had to shoulder the impact of the changes as the PwMND was unable to share this load. Even after learning about symptoms, some carers felt guilty and disloyal to the pwMND by having to talk about these difficult behaviours, even if the PwMND was not present in the room.

*“Well I suppose I was feeling guilty about sort of sharing with other people, you know, people like yourself or whoever is involved in it, sharing negative things about him.”****Carer 09***.

Carers reported sadness and loss of the person that they used to be, not only in relation to physical abilities, but to their cognitive abilities, which they found the most difficult.

*“And I think it’s the psychological side of the condition that I find hardest to cope with. The physical side, I mean to a great extent you can get your way around a lot of the physical, although obviously it will get harder. But a lot of the mental stuff is quite difficult, it is quite challenging […] And it’s just that you realise it’s not – although it’s the same person, some of the sharp comments or different things aren’t the comments that you would have had previously.”****Carer 10***.

BehSymp negatively impacted on carers, who reported that this was not what they had expected of a terminal illness. The unnamed experiences of BehSymp also led to other negative feelings such as pain and anger.

*“Well it was kind of like unknown territory really. You know, you’re dealing with someone that you know very well but behaves in a way that you know is not their usual behaviour. And also as well, you know, [name 2] is someone who I care for and I love who has got a terminal illness, so there is guilt, there is anger, there is all of those emotions all rolled up. And then you’re dealing with someone who at times seems unreasonable and you can’t respond in the normal way you would do, so confusion […] Confusion and anger and upset and all those emotions, they’re all – it’s a very jumbled, you go from one to the other. It’s like there are three emotions, you jump from one to the other and then you think in between and then you have to approach the whole thing a different way, each time, yeah.”****Carer 01***.

Relationships became strained as some carers interpreted the PwMND’s behaviour as intended actions towards them, not symptoms. For example, carers reported that the PwMND had become unreasonable and selfish, when they may have lost their ability to empathise and read other people’s emotions. Disappointment was also reported, especially when lack of empathy was present.

*“You know, being unreasonable, being selfish to the point of not understanding. Because we’re all selfish but we understand when we’re being selfish. But when someone is being totally selfish and don’t understand it, that’s really hard, that’s a hard one to deal with when you want things your own way all the time or you want things done now because that’s what you need, your need must be met straightaway, regardless of whatever else I’m doing or involved with, you know?”****Carer 01***.

### Theme 2. Others can see and support: the role of HCPs in identifying behavioural symptoms - and perceived opportunities for carers to receive support

#### Professional-led identification of behavioural symptoms and support

Carers related several instances where the identification of behavioural symptoms were led by a member of the MDT. These were either directly via a conversation, after the result of a cognitive assessment, or triggered by genetic testing which would have indicated that the MND phenotype was related to a genetic form associated with frontotemporal dementia.

*“Because the motor neurone disease nurses had made me aware of what the frontotemporal dementia would bring, how it would manifest itself. So you know, when you hear that and then you start putting things together, you think ‘OK, yes’.”****Carer 06***.

*“Yes. Well as the neurologist explained to me, she said, it’s like, she said there are lots of things which are still there. She said, ‘Look, with [PwMND]’, she said, ‘It’s a frontal’, and she said to me, ‘It’s like the captain of the ship has left so everything is a bit of a muddle’.”****Carer 01***.

Against the difficult context of a rapidly progressive condition such as MND, carers reported that without intervention from the professionals, they may not have realised that they needed help with non-motor symptoms or expressed uncertainty as to which type of support they required. Carers were therefore particularly positive about instances where professionals helped them understand behavioural symptoms and stepped in to provide support for carers. Some professionals highlighted that the carer needed support and referred to counselling or took the time to explain the behavioural symptoms in MNDFTD.

*“When the dietician, as I said, she rings every five weeks I think it is, and I always answer the phone and she always says, “How is it going [carer’s name]? How is [PwMND}, has he been good?” And you know, “Have you tried that?” I said, “He’s still losing weight.” “Have you tried this? Have you tried that?” That’s really helpful to me.”****Carer 05***.

#### Services are not designed to support carers

Despite some positive experiences with HCPs described above, many carers struggled with lack of support. Carers reported not having the experience of being told by HCPs that non-motor symptoms were part of MND, prior to the MiNDToolkit. Carers alluded to the limited opportunity for open discussion with professionals in the current service design; with some carers feeling excluded from the appointment. At times, communication played a big role in misunderstandings, with carers feeling that they were ‘abandoned’ by the team. Telephone contact was also not viewed as proper support, being deemed as too impersonal.

*“And I think the other thing is, is that whatever is told to you in clinic, you only – you don’t, a) it’s all about the patient, not the carer, and also […] it’s very much, it’s from a clinical perspective, so it’s the medical side of the condition, not, just not so much the psychological side of the condition. And I think it’s the psychological side of the condition that I find hardest to cope with. The physical side, I mean to a great extent you can get your way around a lot of the physical, although obviously it will get harder. But a lot of the mental stuff is quite difficult, it is quite challenging.”****Carer 10***.

Carers felt that current services were focused only on time with the PwMND, with some carers even reporting that they did not feel they had a right to receive support from the NHS and felt that they should be coping on their own. Even when professionals appropriately recognised the carer’s need for counselling, and this was set up, it still left the carer feeling ‘like a fraud’ for having received help.

*“’Just phone me any time’. but I just know how busy these people are [HCPs] and I just feel I should be able to cope with this on my own, so I don’t contact them. I know that for the NHS to provide help for carers, they haven’t got the funds to do that, so it’s a very difficult call but I really think there’s a need and perhaps the coffee morning will be our answer if we try it again because that’s not financially dependent on the NHS, that’s MND Association that does that.”****Carer 05***.

Outside of NHS services, some carers reported receiving informal support from volunteers from the MND Association, family and friends, which occurred alongside formal support. But for others, these informal channels felt like their only source of support. Thinking ahead, some carers decided to take part in research because it would offer them an opportunity to receive greater support from their MND team.

*“But just even if it was for me to pick up the phone to one of the other carers and say, “This is happening, you know, is this normal?” I feel that I could probably do that rather than pick up the phone and speak to somebody like [HCP], who I know are really, really busy people. If it’s another carer I think you can be a bit more informal and, I don’t know.”****Carer 05***.

*“From a very selfish perspective, felt that if we were actually involved [in research] we’d probably get more help because we would know the people more and feel – if you’re involved, we felt we’d be able to ask for help more than if you’re – if all you’re doing is going along to an appointment every four months, you have no relationship.”****Carer 10***.

### Theme 3: Carer implementation and perceived impact of the MiNDToolkit content

#### The full picture of MND resonating with carers

Carers reported that, prior to MiNDToolkit, they thought MND to be a ‘physical disease’. As such, behaviour symptoms were perceived to be out of context. This gap in understanding often led to carers attempting to rationalise with the PwMND, which lead to arguments.

*“I found it really, really hard to be honest because the natural reaction is to try and explain to the person, ‘No, you’re wrong, you know, we haven’t got bedbugs and they haven’t – the neighbours haven’t stolen our land.’ And it ends up in an argument because you can’t kind of explain to them that what they’re thinking isn’t right. And it’s only since doing the MiNDToolkit that I’ve kind of realised it’s not worth trying to correct them. Just, you know, just accept what they’re saying and just maybe distract, we change the subject.”****Carer 02***.

With MiNDToolkit, carers acquired understanding that those BehSymp were due to MND, which in turn alleviated the uncertainty of the root cause of the distressing behaviours. Carers were able to put together what they had observed, with the information provided by their nurse, and felt it ‘all makes sense’. Additionally, they felt that MiNDToolkit gave legitimacy to what was happening, because there was scientific evidence in explaining the difference between apathy and depression.

One of the carers found MiNDToolkit resonated particularly well with them, given that the PwMND they cared for had had BehSymp due to a pre-existing condition.

*“Oh crikey, where do I start? It was like a light bulb had gone off, literally and there was so many things as I was going through the modules and I was thinking, “Wow, yes, that’s happened to me, that’s happened to us.” And, like I say, it was a real revelation to know that all these things that we’d been going through were actually part of the disease and it wasn’t my partner becoming more obstructive, more awkward, more combative, as something in addition to MND, it was actually part of MND. And, yeah, and the hallucinations as well, you know, it kind of all made sense and it all fitted into place, yeah.”****Carer 02***.

Carers felt empowered to be able to talk about symptoms, using new terms learned via the platform. A sense of control could also be noticed when carers felt prepared for potential symptoms in future.

*“They were helpful because I hadn’t – at that stage, the very fact I could talk to you about it, I wouldn’t have been able to talk to you about it if I hadn’t watched the video because I wouldn’t have known.”****Carer 10***.

#### Full picture leading to validation and acceptance

The MiNDToolkit also validated carers’ experiences. Videos from the modules provided reassurance that other families have had similar struggles, and that options for help were available. Validation of their emotions supported normalisation of their reactions, while reassuring carers that they were doing the best that they could.

*“I think to know that other people have struggled the same as I’m struggling, that it’s not a weakness that I’ve got, other people are struggling with the MND, with the illness itself, and the people that they love that have got the illness. That there is, when they get, when the person with MND gets really bad, there is help there if needed”.****Carer 05***.

Increased knowledge and understanding about BehSymp appeared to facilitate acceptance of them as part of MND, which leads to changes in carer’s own behaviours and reactions, as well as using strategies, when BehSymp occur. Acceptance of BehSymp was also demonstrated by carers reflecting that the PwMND cannot help behaving in a new certain way, that behaviours may not be rational choices, and that their new approach is to use the learned strategies rather than arguing back.

*“Well now I just accept that he does have these thoughts and I don’t get upset with him myself, which I would have done previously. And I don’t contradict him, I don’t argue back, I just accept what he’s saying and kind fall short of doing any – offering to do anything about it because obviously I can’t do anything about all these things that he’s thinking. But I will try and - I’ll try and change the subject onto something else.”****Carer 02***.

#### A new approach: carer able to reduce conflict, feeling upskilled and sharing new learning

Strategies suggested by the MiNDToolkit added new skills to carers’ portfolios, making them feel supported and equipped. This learning enabled different actions and reactions, making things easier for the carer by reducing conflict. Carers also learned to phrase things differently, to reduce resistance from the PwMND in decision making processes.

*“I think the main thing is that you suggest in the Toolkit, this is just strategies that you can employ. And I think having those strategies as a kind of crutch, something to lean on, something to look up, it kind of makes you feel that you are supported in a way because you’ve got a way of dealing with it. Rather than, you know, if you’re just on your own, you just kind of look at that person and you’re dealing with them as though it’s somebody who hasn’t got any behavioural issues and there’s a tendency to kind of react in the moment whereas, you know, if you’ve got that Toolkit, you can look at various different ways that you could react.”****Carer 02***.

But some strategies were not always applied as intended by MiNDToolkit. A couple of examples demonstrated that carers may have understood that a certain behaviour reflected a symptom, e.g., ‘rigidity/lack of flexibility’ (part of MiNDToolkit) - but then applied a strategy of ‘shouting’ to try to overcome the symptom of rigidity (not part of MiNDToolkit). Most of the time, though, carers demonstrated application of the learned strategies during interviews, for example ‘Prepare and Increase Awareness’, through sharing the learning with others.

*“And the other thing which I’ve started to do is, yeah, is to tell friends and family about the changes beforehand […] To tell, let everyone know beforehand that there is a slight change in personality. So I wasn’t feeling the pressure it brought, so in social occasion.”****Carer 01***.

### Theme 4: acceptability and carers’ engagement with MiNDToolkit

We identified some key factors in carers’ engagement with the intervention.

####  Timing for using MiNDToolkit

For some, the key motivation to engage with the MiNDToolkit was a desire to prepare for future symptoms and/or a need to understand the causes of behavioural changes. For these carers, early access to the MiNDToolkit was important to equip them to deal more confidently, and better, with the behavioural changes associated with MND.

*“I know some people think ignorance is bliss, but I like to know more information so that I’m prepared. So for me, personally, I would rather know well this might happen or this could happen. And just knowing that it may happen then I can prepare myself.”****Carer 05***.

Some carers felt MiNDToolkit arrived too late, when they had already learned to adapt to the situation. As such, most carers reported that accessing MiNDToolkit early in the post-diagnostic phase would have been more useful. However, others felt an early introduction might be overwhelming whilst they were still coming to terms with the diagnosis. A lack of readiness was generally associated with anxiety and fears. Some carers reported anxiety in knowing about things that might happen, which at times led to avoidance in engaging with the platform. Others reported that they knew they needed to engage with MiNDToolkit, but felt they needed to be in an emotionally stable place to take things in.

*“I think that maybe about a month after diagnosis because I know at the time [PwMND] was diagnosed, I was on the internet, I was looking through the book and reading up as much as I possibly could about it to know what we could expect in the future and how it might progress. But then as time wears on you kind of put all that to the back of your mind and just focus on coping. So I think at some point initially, maybe within the first couple of months, the Toolkit might – it might be good then to introduce it whilst somebody is still in the frame of mind that they want to know as much as they possibly can and at least then they’re prepared for the behavioural and mental changes ahead of time. You know, they know about it from more or less the get-go.”****Carer 02***.

Overall, it appeared that a gentle introduction by HCPs, responding to the carers’ cues would be the most appropriate guidance for the timing. For some carers with initial perceptions that they did not need support from the MiNDToolkit, timely intervention and encouragement from trained HCPs was highlighted as vital for engagement.

*“Initially when I just got the information through the post saying, “Do you want to do this?” My initial reaction was no, partly because I thought can I be bothered? Do I need to do it? I wouldn’t have done it had the OT – had we not had that discussion about him being fixated on this bit of equipment and her saying to me, you know, it was just, I suppose fortuitous that it happened at the same time as I’d received the information about it, you know. And she said to me, “Have you received the information about this thing? Do you want to do it?” And I said, “Yes, I have but no, I probably won’t do it.” And she said, “I absolutely think you should because it will help you understand why he’s behaving like this. So you know, until that point, if we hadn’t had that conversation, I possibly wouldn’t have done it.”****Carer 09***.

The stage of the disease itself was also factor in the timing for carers’ engagement. Progression of the disease whilst having access to the MiNDToolkit was seen as an enabler as carer felt they had more experience and related better with the content (carer who continued with intervention after end of the study). For others, the increased disability and greater needs of the PwMND meant that carers could not engage with MiNDToolkit due to reduced time. Other external factors played a role in engagement, such as carers’ own health state or other family members’ needs.

*“So there was the first lot of modules that I did over three months, which actually I didn’t quite get to the end of because various other things were happening. I had my own health issues and everything and so I didn’t quite get to the end. And then suddenly the three months was up.”****Carer 09***.

#### Using the platform - ease of use

Carers found the platform modules to be accessible as they were short, could be paused, and could be fit around other duties, including nighttime hours. The platform was felt to be straightforward, and reminders were useful for continued engagement. Its online nature was also seen as a positive aspect for repeating things, and for continued access.

*“Whereas the MiNDToolkit, it was in nice, easy, bite size portions, if you like, to coin a phrase. But yeah, nothing took more than sort of like 10,15 minutes which was great for me. So I could do in between, you know, household jobs or I could even access it at work and do a couple of modules during my lunch hour or in a break. So it’s that kind of, you know, short, only about five, 10 minutes long which makes it more accessible I think”.****Carer 02***.

*“But as I say, I have now done it all and some of it several times, so yeah, it was certainly worth doing so I’m pleased I did it. I was very glad to get the opportunity to do it again, or to go back and revisit it, and I certainly found it beneficial because there was, as I say, well all the cognitive changes were things that I didn’t know about and wasn’t expecting. And although I had noticed changes, I certainly, at that point, hadn’t connected them or hadn’t realised other people did that sort of thing as well, you know.”****Carer 09***.

Some carers took the intervention into new directions that had not been intended at the conceptual phase. For example, they shared their login with their children, or even showed the content to the PwMND.

*“So I gave it to him and said, “Look, this is,” because he couldn’t understand why things were happening. And I said, “Well look, this is why this is happening, look at this three-minute video on empathy and apathy.” And he watched it and felt so much better because he had seen it actually explained to him.”****Carer 10***.

*“Because I sort of thought it might be useful for my son to have a look at it because that’s what I – yeah […] you know, the explanations I thought maybe would be useful for him, I could get extra, yeah.”****Carer 04***.

#### Reinforcement by HCPs

Carers perceptions of their need for reinforcement by HCPs were varied. Those who received reinforcement seem to have appreciated it coming from a professional with whom they already had a relationship of trust, or whose background training were more fitting in the carers’ view. Some who did not receive reinforcement wished they had had, while others reported that they do not think they would have needed it.

*“I’ve discussed it with are the Occupational Therapist and the Neurology Nurse. They’re probably the only two people I have talked to about it and they have ex – you know they come here and see him, they’ve experienced some of his behaviour. I suppose with other people, I don’t know, like Speech Therapist, Physio, etc., they see him as a patient and I’m normally there, so it’s not a situation where we’re sitting having a discussion about, you know, it hasn’t come up. It’s not part of their remit anyway I suppose, you know, they’re doing this, whatever they do. So it’s not that I’ve chosen not to discuss it with them, it’s just not been appropriate.”****Carer 09***.

### Theme 5: future implementation

All carers interviewed had suggestions for improving MiNDToolkit, demonstrating strong engagement and keen interest in promoting access to others in future.

#### MiNDToolkit content

Carers had excellent suggestions for new modules that could be added. These included information on emotional lability, changes in relationships, and transitioning to a care home. One carer suggested including more explicit examples applicable to PwMND with bulbar symptoms.

#### Delivery/Hybrid

Carers were unanimous that the delivery of MiNDToolkit should be maintained as hybrid, combining online and face-to-face learning from HCPs, but suggested that reinforcement could be an option based on the carer’s needs and wishes.

*“I think initially online was fine because […] when you do it online, you can do what you’ve got time for and then go back, whereas if you were face-to-face you wouldn’t have that option […] But I think perhaps do it in two parts, do part online and part face-to-face”.****Carer 05***.

#### Expansion, and what did not work so well

Carers wanted to encourage other carers to use MiNDToolkit in future. They also wondered if volunteers could be involved in MiNDToolkit, through reinforcement.

*“I mean I would certainly encourage other people to do it. And sort of whether it’s worth trying to encourage the medical, the health professionals to encourage carers as well as just sending out a paper, you know, invitation.“****Carer 09***.

But not all elements of MiNDToolkit were well accepted. Some carers felt that they were not IT savvy and would have preferred an HCP-based delivery, with paper resources. Others would have liked the flexibility to navigate the modules as they wished – the delivery was offered in a certain order. The flexible navigation can be set and taken forward in the next MiNDToolkit study.

## Discussion

Our study confirmed that carers’ experience of BehSymp can be emotionally distressing, in particular when out of context and perceived as personal reactions - and not part of MND symptomatology. By using MiNDToolkit, however, carers reported learning and making sense of BehSymp, understanding that the full picture of MND goes beyond motor deficits. This process supported acceptance and management of BehSymp and was appreciated when accompanied by HCP support – even though some carers interacted with the MiNDToolkit intervention solely via the bespoke platform.

Carers’ challenges in dealing with BehSymp in MND are compounded. Carers not only have to deal with known triggers of emotional distress in MND, such as a threatened future and keeping up with multiple changes [23], but they also experience behaviours from the PwMND which are not consistent with the person they know [24], as shown in theme one. In our study, rigid and obsessive behaviours made routines inflexible, disinhibition caused embarrassment, and lack of flexibility led to conflict between the carer and the PwMND. Relationships appear to become strained, with greater sense of loss [25]. Indeed, our findings align with other studies where BehSymp were identified as key factors influencing carer burden [12–15], and should be clear targets for intervention.

The importance of having a MND professional involved in an intervention such as MiNDToolkit was highlighted by the majority of carers, even by those who did not discuss MiNDToolkit content in detail. HCP involvement appeared to lead to greater validation of the intervention, as shown in theme two, making it legitimate – especially in the context of other online/social media resources, where some carers feel that these may not be trustworthy. It is interesting, however, that some HCPs involved in the study worried that highlighting challenges with behavioural symptoms would be burdensome to carers [18].

Having HCP involvement in the programme also seemed to strengthen the connection of the carer with the MND service involved with the PwMND who they provide care for. As services are mostly focused on the PwMND, as highlighted in theme two, carer engagement with MiNDToolkit opened or reinforced a connection between the HCP and the carer, creating safe spaces for carers to discuss their own concerns and that may not normally feature in regular MND appointments. This triangulation seemed to be valuable to carers. Indeed, other MND studies showed overwhelming positive responses from carers [26] in engaging with therapies and HCPs in communicating [27] well and supporting their experience of caring for a PwMND [23]. Future service design should take carers’ needs into consideration.

In exploring the experiences of carers receiving MiNDToolkit, this present study revealed that carer self-efficacy is the area to be targeted in the future large trial. Theme three exposed the process of carers moving from a position of thinking that MND was only a physical disease, to a position to understanding the full picture of MND. This led to validation of their lived experience of dealing with behavioural symptoms and acceptance of these symptoms as part of the disease. As such, they were able to try out new ways to manage behavioural symptoms, reducing conflict and sharing new learning with others around them. In the future trial, a measure of self-efficacy [28] should be included.

An important aspect of the psychoeducational intervention was the involvement of the trained HCP, which varied per site [18], impacting on the MiNDToolkit implementation as shown in theme four Carers unanimously reported that the intervention should remain hybrid, but not all were certain that they would have wanted regular HCP input – while others would have preferred greater HCP input. It appears that a modulation of HCP involvement would be ideal, with HCPs responding to carers’ need in a dynamic way. Nuanced communications, tailored for the carer, seems to be a good way forward in response to carers’ experiences reported in MiNDToolkit and the scientific literature [27]. HCPs should be reminded that carers place great value on their input, time, and expertise.

MiNDToolkit appeared to be well accepted by most carers, with some expanding its reach by providing the platform login to other family members or even showing modules to the PwMND. While these steps were not intended or described in our research protocol, they seem to suggest that carers found the content helpful. Theme four uncovered that the MiNDToolkit platform offered great accessibility, confirmed through unusual hours of use, e.g., very early or very late in the day. Unlimited online access seems to encourage carer participation, as it offers flexibility and carer control; this enhanced online accessibility has also been shown in other online interventions for carers [29, 30]. As such, future interventions targeting carers of pwMND should be designed with flexibility and accessibility in mind. Delivery of content could combine paper, online resources, and human contact, thus ensuring inclusion of a wide range of carers’ preferences and accessibility needs.

Carers were also keen to provide suggestions on how to enhance the MiNDToolkit, as shown in theme five - most noticeably in relation to the role of the HCP as discussed earlier. Other more practical suggestions were also mentioned. For example, carers asked for new modules on mood lability, relationship changes, and transitions in care, as well as an accompanying paper version of the content to increase accessibility and as a memory aid, similar to HCPs’ request [18]. These can be addressed for a future trial.

Limitations of our study include its online nature. As MiNDToolkit was transformed to be a full online intervention because of the COVID pandemic, access was restricted to those willing to engage with an online intervention. In addition, since the study was conducted within the National Health Service in England and Wales only, experiences with HCPs implementing the intervention may not be generalizable. Detailed information on neuropsychological testing on pwMND was not available, as pwMND were not participants in the study. Most carers were female, thus limiting our interpretations of MiNDToolkit’s acceptability by male carers. However, strengths in our study include a diverse ethnic cohort, and participation high completion rates.

In summary, the MiNDToolkit psychoeducational intervention seems to be acceptable by carers of PwMND with additional behavioural symptoms. The intervention helped carers expand their understanding of MND, supporting acceptance of symptoms and enabling carers to modify their own behaviour and apply newly learned strategies.

## Data Availability

Due to the sensitive nature of the interview data, interviews have not been deposited in public archives. Please contact the corresponding author if interested in developing research collaborations.

## Appendix 1: interview guide – MiNDToolkit

*****Note that this topic guide is meant to serve as a starting point which may be amended and adjusted as the themes of the qualitative interviews develop.

1. **Welcome**.

*Introduce yourself and thank the participant for agreeing to take part in the interview.*

2. **Introduction**.

The purpose of today’s interview is to hear about your experiences of using the MiNDToolkit platform and going through the modules with the healthcare practitioner. We’re interested in hearing about your personal views and experiences - there are no right or wrong answers. I have a list of suggested questions to remind me of the kind of things we might want to talk about but exactly what we cover is up to you. Feel free to go into as much detail as you feel comfortable with. If you don’t feel comfortable talking about something then that’s absolutely fine, just let me know – we can change the topic, take a break or stop the discussion at any time. Do you have any questions before we get started?”

*Answer any questions the participant has. Check that they are comfortable and let them know before turning on the recorder.*

3. **Background**.

- Could you please start by telling me your name, and the name of the team that looks after the person with Motor Neurone Disease who you care for?

**Opening question**.

b) **Just to give me a bit of a background, could you tell me a bit about your understanding and experiences with symptoms of Motor Neurone Disease (MND**) **before signing up to the MiNDToolkit study**.

*Prompt for*:

- *Were you aware that people with MND can develop both physical and behavioural symptoms?*
- *What were the main symptoms that x was experiencing?*
- *What was your understanding of behavioural/personality symptoms?*
- *What was your main source of information?*
- *What help you were receiving prior to involvement in the MiNDToolkit study?*

c) **Could you tell me a bit about your experience with managing the symptoms X was experiencing before the MiNDToolkit study**.

*Prompt for*:

- *What challenges were you having in caring for the person?*
- *Experience with seeking help? Any challenges?*
- *What help you were receiving prior to involvement in the MiNDToolkit study?*

d) **What made you decide to take part in the MiNDToolkit study?**

*Prompt for*:

- *Have you ever been involved with an intervention for carers of people with MND before? If so, what was it about?*
- *In what ways was it similar or different to the MiNDToolkit?*
- *What was your experience of having the MiNDToolkit Online Platform being offered by the MND/Community team? (Positive and negative)*

e) **Could you talk me through your experience of going through the MiNDTookit modules?**

*Prompt for*:

- To what extent were the modules appropriate for you and the person you are caring for?
- Did the modules cover the main challenges they were experiencing at the time?
- Is there a module that you felt was most helpful or memorable to you? Why was it so helpful?
- Could you give an example of a strategy that you have used from that module?
- *How did you go through the modules?*
- What was your experience with the support of HCP and how this worked with the Mind Toolkit online platform?

f) **What was your experience of the MiNDToolkit Online Platform?**

*Prompt for*:

- *How often did you use the platform and how helpful was it?*
- Were there features of the MiNDToolkit Online Platform that worked well and you enjoyed using? If so why?
- Were there features of the MiNDToolkit Online Platform that you did not enjoy or were not helpful? And why?
- What were the main difficulties in using the platform?
- What were the easy aspects?
- What did you think of the format of the MiNDToolkit Online Platform?
  - online nature of the intervention.
  - use of automatic reminders.
  - frequency of sessions/duration of sessions.
  - the modules.
  - animations and videos.
  g) **What were you hoping you would get out of the MiNDToolkit Online Platform? Do you think your expectations/needs were met? Wh**y?

h) **Next steps in the MiNDToolkit**.

- *You asked to continue using the platform for another 3 months, why did you do so? How are you intending to use it?*
- *Would you want to see the MiNDToolkit Online Platform been offered to other carers in the future? Why?*

i) **Close**.

Before we finish, is there anything else about your experiences with using the MiNDToolkit online platform and support from the HCP would be useful for us to know?

*Thank the participants for their time and for sharing their experiences and opinions.*

*Explain what will happen to the interview data and how the findings will be used*.
